# Predictors of unmet need for family planning in Ethiopia 2019: a systematic review and meta analysis

**DOI:** 10.1186/s13690-020-00483-2

**Published:** 2020-10-16

**Authors:** Temesgen Getaneh, Ayenew Negesse, Getenet Dessie, Melaku Desta, Tebabere Moltot

**Affiliations:** 1 Department of Midwifery, College of Health Science, Debre Markos University, P.O. Box 269, Debre Markos, Ethiopia; 2 Department of Human Nutrition and Food Sciences, College of Health Science, Debre Markos University, Debre Markos, Ethiopia; 3 Center of excellence in Human Nutrition, School of Human Nutrition, Food Science and Technology, Hawassa University, Hawassa, Ethiopia; 4 Department of Nursing, College of Medicine and Health Science, Bahir Dar University, Bahir Dar, Ethiopia

**Keywords:** Sexual and reprodcutive helath, Birth control, Contraception, Family planning, Ethiopia

## Abstract

**Background:**

unmet need for family planning is a common cause of uninteded pregnancy which mostly end up with abortion. Many studies were conducted on predictors of unmet need of family planning in Ethiopia. But, up until now, single evidence has not been synthesized and various point prevalence estimates of unmet need for family planning have been reported. Therefore, this sytematic review and meta analysis was established to identify the predictors of unmet need for family planning in Ethiopia.

**Methods:**

search engines including PubMed, Embase, CINAHL, Google Scholar, HINARI portal, and Cochrane Library were used to retrieve included articles and reported using the preferred Reporting Items for Systematic Reviews and Meta-Analyses Protocols (PRISMA) checklist guidelines. Joanna Briggs Institute Meta-Analysis of Statistics Assessment and Review Instrument (JBI-MAStARI) was applied for critical appraisal. All observational studies done on reproductive age women and reported on unmet need for family planning were included. Unmet need for family planning is the percentage of women of reproductive age, either married or in a union, who have an unmet need for family planning to stop or delay childbearing. Random effect model was done to estimate the pooled prevalence of unmet need for family planning. Odds ratio with 95% confidence interval was considered to determine the association of identified variables with unmet need of family planning. Cochran’s Q statistic, Egger’s and Begg’s test were carried out to assess heterogeneity and publication bias.

**Results:**

Fifteen articles and 17, 585 reproductive aged women were included to estimate the pooled prevalence of unmet need for family planning and its predictors in Ethiopia. The prevalence of unmet need for family planning in Ethiopia ranges from 26.52 to 36.39%. Age at first marriage < 18 yrs. with OR = 2.3 (95% CI: 1.08, 4.87), women with no formal education with OR = 1.9 (95%CI: 1.19, 3.04), partner with no formal education with OR = 1.78 (95%CI: 1.18, 2.68) and absence of discussion with their partner about family planning with OR = 3.52 (95%CI, 2.56, 4.87) were predictors of unmet need of family planning in Ethiopia.

**Conclusion:**

This meta analysis revealed that, the prevalence of unmet need for family planning in Ethiopia was high as compared with the United Nations sphere standard of unmet need for planning, considered to be high if it is greater than 25%. Early marriage, no formal eduaction and lack of discussion with partner on family planning were predictors of unmet need for family planning. Therefore, efforts are needed to empower women through eduaction, avoiding early marriage and facilitating dicussion of partners about family planning in order to improve family planning usage.

## Background

Unmet need for family planning is the percentage of women of reproductive age, either married or in a union, who have an unmet need for family planning to stop or delay childbearing [1]. It shows the gap between childbearing desires and contraception use and taken as useful indicator towards the target of achieving universal access to reproductive health [2]. At the beginning, family planning was planned as a program to alleviate environmental, economic and societal impact of rapid population growth [3]. But later on, family planning advocated to help women and men to achieve their preference children and to have wanted children when they want them. In addition, family planning also play a major role in maternal mortality reduction [4].

The United States Food and Drug Administration has approved a wide range of modern contraceptives including emergency contraceptives methods for preventing unintended pregnancy. But, unintended or unplanned pregnancy is still the major incapacitating problem which affects millions women and their families worldwide particularly, in low and middle income countries [5]. In 2017, around 800 million reproductive aged pregnant women in low and middle income countries wanted to avoid pregnancy from which an estimated 214 million women have an unmet need for family planning services [6]. Globally, 43% of unintended pregnancies occurred in low and middle income countries in which 74% of them were related to unmet need for family planning. In adding to this, in East Africa unmet need for family planning responsible for 86% of unintended pregnancies [7].

Abortion is a frequent consequence of unintended pregnancy. An estimated of 18 million unsafe abortions take place each year in the low and middle income countries which in turn result in serious, long-term negative health effects including infertility and maternal death [8]. Fully meeting the unmet need for modern contraception would result in an estimated 76,000 fewer maternal deaths each year worldwide [9].

Although women are the primary focus of most of the services offered, unmet need also has an impact on individual, interpersonal, familial and societal at large as well has ramifications for healthy births and babies. Women with unmet need for spacing and limiting has elevated risk of under-five mortality [10]. Although unmet need are related to complex factors, range of constraints can prevent women from family planning service like demographic, socioeconomic factors and proximate factors [11–13].

Sub Saharan Africa has women with the highest number of unmet need for family planning as evidenced by 25% of reproductive aged married women has unmet need for family planning [14]. So, this is the reason why it share the highest burden of maternal mortality related to unwanted pregnancy and unsafe abortion than other regions.

Modern contraceptive use by currently married Ethiopian women has steadily increased. But, still 22% of currently married women have an unmet need for family planning [15]. In Ethiopia, different studies were conducted to assess prevalence of unmet need and predictors [16–30]. The reports of these fragmented studies revealed a wide variability of prevalence of unmet need for family planning with the highest prevalence reported in South Nation and Nationality People region (SNNPR) (52.4%) [25] while the lowest reported in Amhara region (17.4%) [23]. Those studies also addressed factors associated with the prevalence of unmet need for family planning; age, age at marriage, female and male education, discussion with partner and health care provider on family planning and occupation were considered as a common predictors to unmet need.

So, these studies finding in Ethiopia about prevalence of unmet need for family planning and its associated factors indicated inconsistent results, since individual articles were done in different socio-demographic areas with different period of time. Moreover, there was also real differences in terms of prevalence and its predictor variables across the study settings too. Therefore, the aim of this meta-analysis and systematic review was to synthesize the events from previous study and attempt to provide a pooled estimate of unmet need for family planning and identify the predictors in Ethiopia.

## Methods

### Search strategies

This systematic review and meta-analysis was reported using the Preferred Reporting Items for Systematic Reviews and Meta-Analyses Protocols (PRISMA) checklist guidelines [31] (see additional file 1). Published available studies were searched using international data bases; PubMed, EMBASE, CINAHL, Google Scholar, Google and Cochrane Library. In addition, the cross references (lists of already identified articles references), academic and governmental institution online library, grey literature available on local shelves and organization websites were also used to access un-published articles. All studies conducted till June 10, 2019 was included for this review. “Prevalence of unmet need for family planning OR factors associated with unmet need for family planning AND Ethiopia” were used as a key word to search (see Additional file 2). Endnote citation manager software version X7 for Windows was utilized to collect and organize search outcomes and to remove duplicated articles.

### Inclusion criteria

All studies conducted in all regional states of Ethiopia on reproductive aged women and reporting about unmet need for family planning were included in this review. No restriction was applied to language, study setting and study design. In addition, all studies included in this review defined the outcomes of variable as the percentage of women of reproductive aged, either married or in union, who have unmet need for family planning to stop or delay childbearing.

### Exclusion criteria

All identified studies title and abstract were screened for eligibility criteria of the review by reviewers independently. According to selection criteria, full texts of eligible studies were examined. Those papers which did not fully accessed at the time of our search process were excluded after contact was attempted with the principal investigator through email at least three times. Finally, after reviewing their full texts, studies which did not report our outcome of interest and studies with poor quality as per settled criteria of reviewing the articles were excluded from the final analysis.

### Quality assessment

Joanna Briggs Institute Meta-Analysis of Statistics Assessment and Review Instrument (JBI-MAStARI) [32] was applied for critical appraisal of included studies before data extraction. Random selection of the study sample, clear definition of the criteria for the inclusion of the sample in the study, identification and addressing for confounding factors, use of objective criteria to assess the outcome of interest, reliable measurement of outcome variable and use of appropriate statistical analysis method [32] were included in the appraisal tool. After quality assessment, studies which scored five and above out of nine criteria settled by JBI for prevalence studies were included in this review (see additional file 3).

### Data extraction

Two authors completed data extraction using a Joanna Briggs Institute Reviewers’ Manual 2014 [32] with clear inclusion and exclusion criteria. Authors name, region, study setting, study year, study design, sample size, response rate, participant’s age, prevalence and common factors associated with unmet need for family planning were included in the extraction tool. Discrepancies between authors were discussed to reach consensus. For final analysis, the authors considered articles which fulfilled the inclusion criteria.

### Outcome of interest

The primary outcome of this systematic and meta-analysis was to estimate the prevalence of unmet need for family planning. Unmet need for family planning is the percentage of women of reproductive aged, either married or in a union, who have an unmet need for family planning to stop or delay childbearing [1]. The second outcome was predictors of unmet need for family planning. Those factors extracted from included studies were; age of women (< 25 years V_S_ ≥ 25 years), age at marriage (< 18 years V_S_ ≥ 18 years), education (illiterate V_S_ educated), occupation (house wife V_S_ others) and discussion with partner (no discussion V_S_ open partner discussions).

### Heterogeneity and publication bias

Cochran’s Q statistic and inverse variance (I^2^) for quantification with its corresponding p–value using random effect model of analysis were considered to check heterogeneity. I^2^ statistics of 25, 50 and 75% was used to declare low, moderate and high heterogeneity respectively [33]. Heterogeneity was considered when *p*-value less than 0.05. In addition Egger’s and Begg’s tests were done to assess the presence of publication bias, and a p-value less than 0.05 were considered as statistically significance [34, 35].

### Statistical analysis

Microsoft Excel spread sheet format was used to extract the selected articles. Then, extracted data were exported to STATA version 14 software for meta-analysis. For heterogeneity and publication bias assessment, Cochran’s Q statistic and I^2^ (for quantification) and Egger’s and Begg’s tests and its corresponding *p*-value with random effect model (since it minimizes heterogeneity of the included studies [33] were considered. In addition, funnel plot was also done to assess publication bias (see Additional file 4). The pooled prevalence of unmet need for family planning and its 95% CI were presented using forest plots. Factors associated with unmet need for family planning were computed and presented using forest plots with their respect of OR and 95% confidence interval. Subgroup analysis was also conducted by region of the study, study year, sample size and study setting of studies. In addition to this, sample size < 614 and above was considered for subgroup analysis because there was observed difference in the prevalence of unmet need for family planning across the classified sample sizes.

## Results

### Article selection

**Fig. 1:**
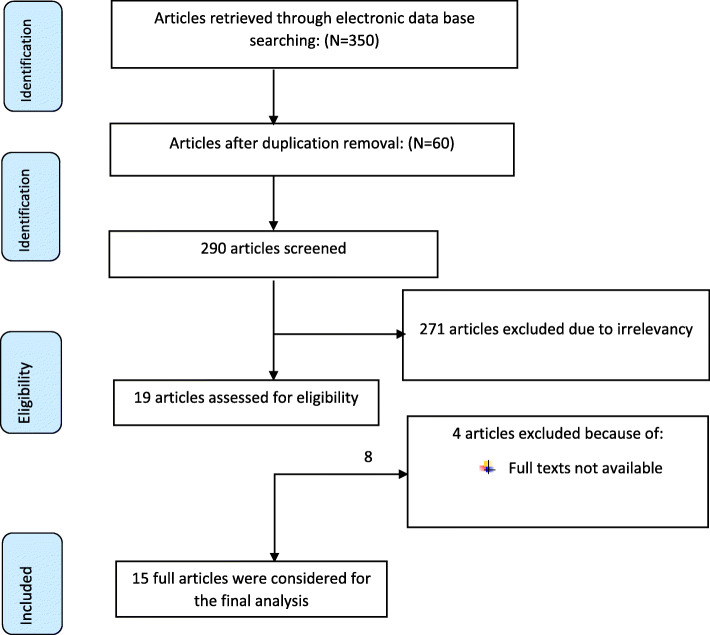
PRISMA flow diagram of included studies to estimate the pooled prevalence of unmet need for family planning and associated factors in Ethiopia 2005–2018

From electronic data base searching, 350 articles related to unmet need for family planning and associated factors were retrieved. From those records, 60 studies were removed after reviewing their titles due to duplication. Then after reviewing titles and abstracts of the remaining articles, 271 articles were excluded because of not related with our outcome of interest. Among the remaining 19 articles, 4 articles [36–39] were removed due to inaccessibility of full articles even after email request till three times. Finally, 15 articles were included for the final review (Fig. 1).

### Characteristics of included articles

A total of 17, 585 reproductive aged women were included in this meta-analysis to estimate the pooled prevalence of unmet need for family planning and predictors in Ethiopia. All studies included were cross sectional and community based except two articles done at institution level [17, 29]. The majority of the included studies were conducted in Amhara regional state [16, 18, 20, 23, 27, 29] and the largest sample size was recorded in SNNPR [25](5746), whereas the smallest in Amhara regional state [29](337). From those studies the lowest prevalence of unmet need for family planning was reported in Amhara regional state [23] while the highest reported in SNNPR [25]. All included articles were conducted from 2009 to 2018 except one study manipulated in 2005 [24]. In addition, almost all studies had a good response rate (≥97%) (Table 1).

**Table 1:** Descriptive summary of fifteen studies included in the meta-analysis of unmet need for family planning and its predictors in Ethiopia 2005–2018

Author name	Study year	Region	Study setting	Sample size	Prevalence	Factors included in each study	JBI score
Biadgie et al. [18]	2015	Amhara	Community	614	23.8	Age, age at marriage, female education, partner education, discussion	7
Gebre et al. [22]	2015	Tigray	Community	510	21.4	Age, age at marriage, female education, discussion with health worker, occupation	7
Dejenu et al. [20]	2013	Amhara	Community	755	25.6	Age, age at marriage, female education, partner education, discussion, occupation	6
Shifa et al. [26]	2010	SNNPR	Community	809	41.5	Age, female education, partner education, discussion, occupation	9
Mota et al. [17]	2012	Oromo	Institution	366	33.3	–	8
Deyessa et al. [21]	2016	AA	Community	2810	22	–	7
Worku et al. [27]	2018	Amhara	Community	411	30.9	discussion, occupation	8
Mekonnen et al. [25]	2009	SNNPR	Community	5746	52.4	partner education,	6
Yibrah et al. [28]	2014	Tigray	Community	1240	31.5		7
Tessema et al. [29]	2013	Amhara	Institution	337	24.3	Age, female education,	6
Genet et al. [23]	2014	Amhara	Community	556	17.4	female education, occupation	6
Chafo et al. [19]	2013	SNNPR	Community	660	26.5	female education, discussion	8
Tegegn et al. [16]	2014	Amhara	Community	383	45.2	Age, female education, discussion,	7
Hailemariam et al. [24]	2005	SNNPR	Community	1988	37.4	–	9
Gebrecherkos et al. [30]	2016	Tigray	Community	400	41.8	–	7

### Prevalence of unmet need for family planning in Ethiopia 2005–2018

From those included studies (15 articles), the estimated pooled prevalence of unmet need for family planning in Ethiopia was 31.45%. Generally, the prevalence of unmet need for family planning in Ethiopia ranges from 26.52 to 36.39%. The lowest prevalence was observed in Amhara regional state [23] (17.4, 95% CI: 11.80, 23.00) while the largest recorded in SNNPR [25] (52.4, 95%CI: 44.64, 60.16).

**Fig. 2:**
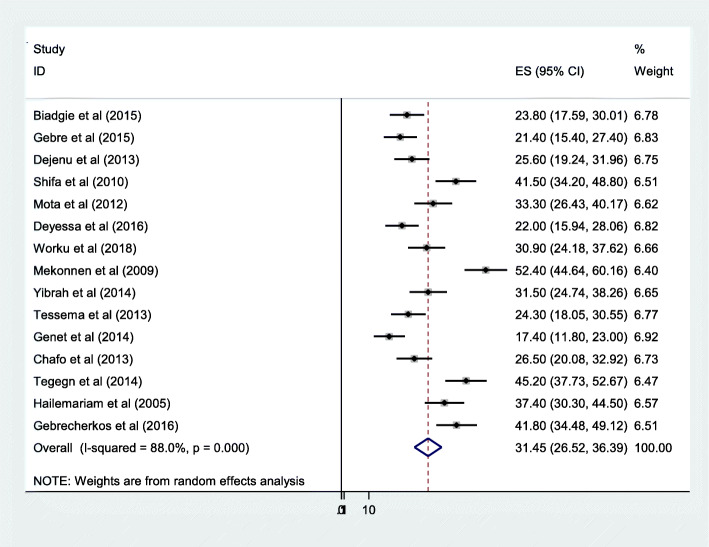
Forest plot of the pooled prevalence of unmet need for family planning in Ethiopia 2005–2018

In Fig. 2, the included studies showed substantial heterogeneity (I^2^ = 88, *P* = 0.000), indicated to compute random effect model meta-analysis and to use prediction interval for interpretation. In addition, symmetric funnel plot, Egger’s and Begg’s tests were done to assess the presence of publication bias. In this systematic review and meta-analysis, both symmetric funnel plot (see additional file 4) and Egger’s and Begg’s test evidenced no publication bias observed (Egger’s and Begg’s test *p*-value =0.458 and *p* = 0.258 respectively).

### Sub group analysis

Subgroup analysis was performed using different study characteristics. The subgroup analysis by region showed that the highest prevalence of unmet need for family planning in SNNPR, 39.5% (95%CI: 28.15, 50.85) and the lowest in others (include Oromo and AA), 27.40% (95%CI: 16.34, 38.47). Whereas, the prevalence of unmet need for family planning in Amhara and Tigray regional state was 27.7% (95%CI: 21, 34) and 31.44% (95%CI: 21, 41) respectively. The prevalence of unmet need for family planning before the year 2014 was 34.49% (95%CI: 24.92, 44.06) and 29.03% (95%CI: 23.44, 34.62) after 2014. In addition, the prevalence of unmet need for family planning conducted on community based was 32.09% (95%CI: 24.50, 39.68) and 31.65% (95%CI: 19.94, 37.58) among studies conducted at institution based. Regarding to sample size, the prevalence of unmet need for family planning with sample size < 614 was 30.5% (95%CI: 22.73, 38.28), whereas the prevalence of unmet need for family planning with sample size 614 and above was 32.6% (95%CI: 22.73, 42.47) (Table 2).

**Table 2:** Sub group analysis which describes pooled prevalence of unmet need for family planning and its predictors in Ethiopia from 2005 to 2018

Subgroup		No of studies	prevalence (95%ci)	Heterogeneity statistics	I^2^	*p*-value
Region	Amhara	6	27.71 (21,34)	92.23	94.6	< 0.001
	Tigray	3	31.44 (21,41)	46.58	95.7	< 0.001
	SNNPR	4	39.50 (28,50)	292.64	99.0	< 0.001
	Others	2	27.40 (16,38)	19.12	94.8	< 0.001
Study year	Before 2014	7	34.49 (24,44)	522.50	98.9	< 0.001
	2014 & above	8	29.03 (23,34)	181.45	96.1	< 0.001
Study setting	community	13	32.09 (24,39)	1304.73	99.1	< 0.001
	Institution	2	28.76 (19,37)	7.03	85.8	< 0.001
Sample size	< 614	7	30.50 (22,38)	142.37	95.8	< 0.001
	≥614	8	32.60 (22,42)	1085.64	99.4	< 0.001

### Meta regression

In order to identify factors associated with source of heterogeneity of the pooled prevalence of unmet need for family planning, meta-regression was undertaken by considering both continuous and categorical data. Sample size, study year, study setting and study region for each individual studies were considered in the meta-regression. But, the meta-regression showed that the pooled prevalence of unmet need for family planning was not associated with sample size, study year, study setting and region of study (Table 3).

**Table 3:** Meta regression for the included studies to identify source of heterogeneity for the prevalence of unmet need for family planning in Ethiopia from 2005 up to 2018

Variables	Coefficients	***p***-value
Study year	−1.415	0.093
Sample size	0.0032	0.084
Study setting		
Institution based	−3.292	0.689
Community based	Reference	Reference
Region		
Amhara	Reference	Reference
SNNPR	11.768	0.090
Tigray	3.711	0.605
Others	−0.268	0.974

### Predictors of unmet need for family planning

**Fig. 3:**
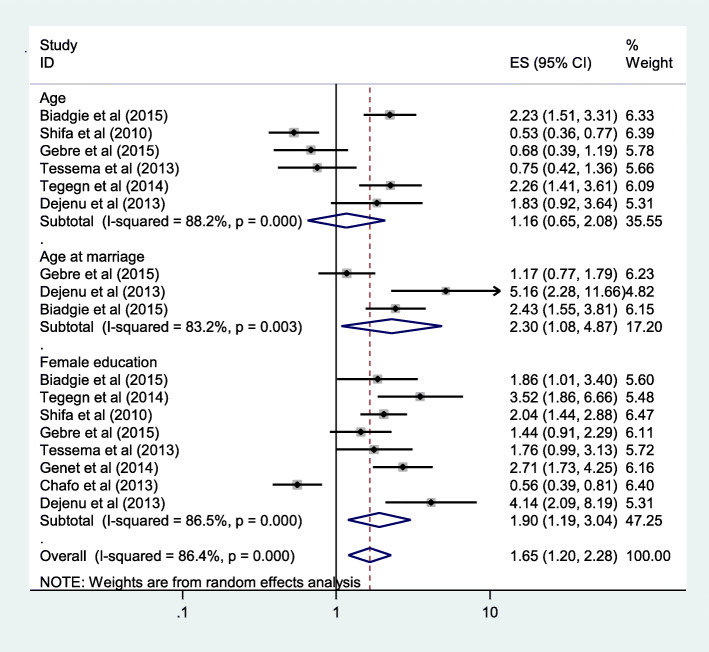
Forest plots which describe association between age of women, age at marriage and women education with unmet need for family planning in Ethiopia 2005–2018

As shown below in Fig. 3, we tried to investigate predictors of unmet need for family planning. Age of the women, age at first marriage, education level of both women and their partner, discussion with partner about family planning, discussion with health care provider about family planning and occupation were predictors assessed for association. But, only age at first marriage, education level (both) and discussion with partner and with health care provider about family planning were significantly associated with the pooled prevalence of unmet need for family planning. Age at first marriage was reported in three articles [18, 20, 22]. Women with age at first marriage < 18 yrs. were 2.3 times more likely to have unmet need for family planning than women marriage at 18 yrs. and above, OR = 2.3 and (95% CI: 1.08, 4.87) (Fig. 3).

Level of female education was another factor recorded in eight articles [16, 18–20, 22, 23, 26, 29]. The odds of unmet need for family planning was 1.9 times higher in illiterate women than literate women (read and write, primary and above educational level), OR = 1.9 and (95%CI: 1.19, 3.04) (Fig. 3).

**Fig. 4:**
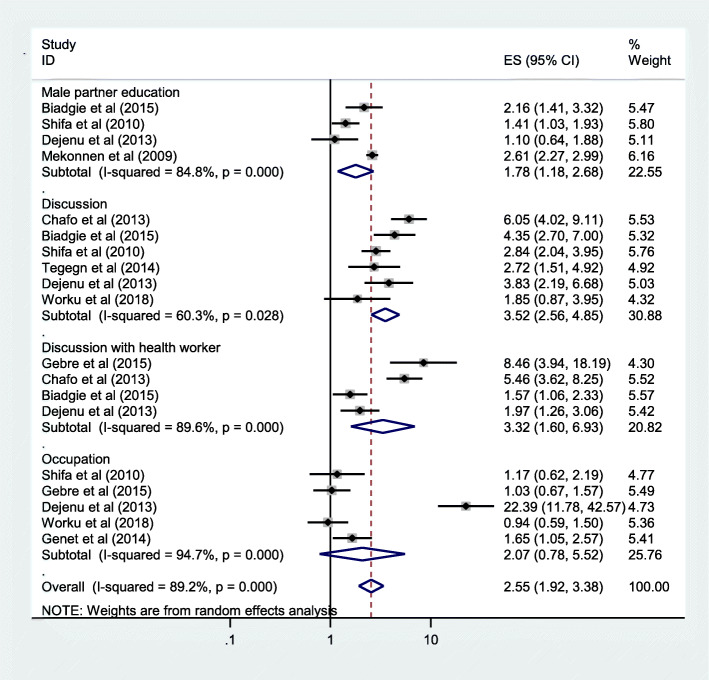
Forest plots which describe association between partner education, discussion and occupation with unmet need for family planning in Ethiopia 2005–2018

The association between male partner education and unmet need for family planning was reported in four articles [18, 20, 25]. The likely hood of unmet need for family planning in women with illiterate male partner was 1.78 times higher than women having literate male partner, OR = 1.78 and (95%CI: 1.18, 2.68). Another factor which was associated with unmet need for family planning was discussion, reported in six original articles [16, 18–20, 26, 27]. Women with no discussion with her partner about family planning were 3.52 times more likely to have unmet need for family planning when compared with women with pertinent discussion with partner, OR = 3.52 and (95%CI: 2.56, 4.87). The last but not the least factor associated with the pooled prevalence of unmet need of family planning was discussion with heath care provider about family planning which was stated in for articles [18–20, 22]. Couples who had no open discussion about family panning with health care provider or community health extension worker had 3.32 times more likely to have unmet need of family planning than those women having open discussion with health care provider or community extension worker about family planning, OR = 3.32 and (95%CI: 1.60, 6.93). The other two factors (age less than 25 yrs. and occupation of women-being house wife) were not significantly associated with unmet need for family planning, OR = 1.16 and (95%CI: 0.65, 2.08) and OR = 2.07 and (95%CI: 0.78, 5.52) respectively (Fig. 4).

## Discussion

Generally, there is great discrepancy among individual articles. For example, study conducted in Bahir Dar revealed that the prevalence of unmet need for family planning was 24.3% [29] whereas study conducted in Arba Minch, Southern Ethiopia reported unmet need for family planning as 41.5% [26]. In addition, study undertaken in North West Ethiopia reported 23.3% [18] unmet need of family planning while article done in Tigray region indicated unmet need of family planning was 41.8% [30].

This systematic review and meta-analysis was computed to estimate the pooled prevalence of unmet need for family planning and its predictors in Ethiopia. The pooled prevalence of unmet need for family planning in Ethiopia was 31.45%. But, the inverse variance test (I^2^) (test of heterogeneity) indicated significant heterogeneity across included studies which was difficult to pooled the prevalence. So, the prevalence of unmet need for family planning in Ethiopia ranges from 26.52 to 36.39% which was higher when compared with SSA report [14]. This higher prevalence of unmet need in Ethiopia could be due to fertility related reasons (breast feeding and postpartum amenorrhea), lack of knowledge on family planning (women had no knowledge of a source for method and didn’t know of a method), opposition to use (either from respondents, husbands or religious prohibition) and health concerns (including side effects) [20, 23, 40].

In addition, the sup-group analysis using time period evidenced that studies done before 2014 showed that 34.5% while 29% of unmet need for family planning among studies done after 2014. This could be due to increased availability and utilization of modern family planning option and governmental and community concern for family planning [41]. Furthermore, there was also prevalence of unmet need for family planning difference across regions. The possible reason may be due difference in socio-demographic characteristics across regions, reproductive health coverage and difference in utilization of family planning in major remote rural areas of regions [42]. To address the issue of unmet need promptly and to identify program options, it is imperative to examine this issues that are responsible for the non-use of family planning methods among women.

This finding is in line with research conducted in Saudi Arabia, which reported the prevalence of unmet need for family planning as 32.6% [43]. In the other hand, our finding was lower than studies conducted in India and Eastern Sudan, showed the prevalence of unmet need for family planning was 40.6 and 44.8% respectively [44, 45]. However, this result was much higher than studies conducted in Egypt and Cameroon, which reported the prevalence of unmet need for family planning as 11.2 and 20.4% [46, 47]. Our meta-analysis also tried to investigate factors associated with unmet need for family planning. Age at marriage < 18 yrs., illiteracy, absence of discussion between partners and absence discussion with health care providers about family planning were the predictors of unmet need for family planning.

Those women married before 18 yrs. were 2.3 times more likely to have unmet need for family planning than women married after 18 yrs. This may be due to adolescent women have less chance to decide number of family size and when to have than adult women. In addition, adolescent women are mostly dependent and less aware about their reproductive issues. So, age is the major determinant for acquiring knowledge and gathering information through different contacting and communication ways (lower age group have restricted communication issues). Therefore, it affects the woman’s ability to make her own decision regarding the reproductive health.

The odds of unmet need for family planning among illiterate women was 1.9 times higher when compared with women having formal education. This finding is in agreement with study conducted in Saudi Arabia and Nigeria, reported as women with no formal education had higher odds of unmet need for family planning [43, 48]. In addition to this, those women with partner illiterate was 1.78 times more likely to have unmet need for family planning than women with educated partners. This is also consistent with finding in Eastern Sudan, lower level of husband’s education negatively affect utilization of family planning [45]. Education is the major tool to increase awareness level and better access for information in family planning so as desire for post-pone fertility. Women should be adequately empowered in education so as to improve knowledge and awareness of methods and sources of contraceptives which has been found as a major barrier to contraceptive use.

This review also showed that discussion with sexual partner was significantly associated with unmet need for family planning. Women who didn’t have discussion with their sexual partner about family planning were 3.52 times more likely to have unmet need for family planning when compared with women having discussion with their sexual partner on family planning service. This association is in line with finding of study done in Cameroon, discussion on family planning within the couple had statistically significant protective association with unmet need for family planning [47]. This may be due to that women who had partner support and clear decision on family planning will have good attitude and initiative to contraceptive. Therefore, women involvement in decision particularly that affect their health status is very important. The other predictor was absence of discussion with health care providers or community health extension workers about family planning. Couples who had no open discussion about family panning with health care providers or community health extension workers had 3.32 times more likely to have unmet need of family planning than those women having open discussion with health care providers or community extension workers about family planning. This finding is evidenced by having open discussion about family planning options, availability, side effects, health related family and country side advantages with health care providers and community health extension workers will have influential positive impact on women’s knowledge, attitude and practice on family planning usage [49]. So, not having such communication will hinder family planning usage and aggravate unmet need of it. This finding was in line with findings of study done in India [50]. As this review reported both the pooled prevalence of unmet need for family planning and its predictors, it is an influential input for health policy makers and program implementers to investigate the gaps on those factors.

### Limitation of the study

This review includes articles reported only in four regions and one administrative city. So, it didn’t include all regions and administrative cities. Those articles included in this systematic review and meta-analysis were conducted in cross sectional study design in which the finding might potentially affected by confounding variables. In addition, the review analyzed only studies reported in English language which might restrict our findings. As we have discussed earlier, I^2^ in this study evidenced the presence of heterogeneity across included studies. Furthermore, in this review variables were classified dichotomously which may in turn vulnerable for much information lost. So, the statistical power to detect a relation between the variable and outcome is reduced. Moreover, in this analysis of predictors we have used crude odds ratio, considering the effect of only one predictor variable. Therefore, it is difficult to getting weighted ratio over strata of co-variables using crude OR. So, unable to control confounding variables. Finally, we like to recommend for researchers to conduct country based studies to assess other confounding factors related to health policy and health service related factors for the prevalent unmet need for family planning in Ethiopia.

## Conclusion

The prevalence of unmet need for family planning in Ethiopia was high. Age at marriage < 18 yrs., illiteracy (both women and their partner) and absence of discussion with their partner were predictors of unmet need for family planning. Therefore, Minister of Health and family guidance with their stake holders should emphasis on community based programs to alleviate those factors leading to this prevalent unmet need for family planning.

## Supplementary information

**Additional file 1.** : Preferred Reporting Items for Systematic Reviews and Meta-Analyses Protocols (PRISMA-P) checklist.

**Additional file 2.** : Searching strategies applied for different searching data bases.

**Additional file 3.** : Joanna Briggs Institute Meta-Analysis of Statistics Assessment and Review Instrument (JBI-MAStARI) critical appraisal of studies.

**Additional file 4.** : Meta funnel presentation of the pooled prevalence of unmet need for family planning, Ethiopia.

## Data Availability

Data will be available from the corresponding author upon reasonable request.

## Abbreviations

AA: Addis Ababa
SNNPR: South Nation and Nationality peoples Region

## References

[1] United Nations**,** Department of Economic and Social Affairs**,** Population Division. World Contraceptive Use 2014.

[2] Alkema L, Kantorova V, Menozzi C, Biddlecom A (2013). National, regional, and global rates and trends in contraceptive prevalence and unmet need for family planning between 1990 and 2015: a systematic and comprehensive analysis. Lancet.

[3] Family Planning Association. A history of family planning services fact sheet. Contraceptive Education Service. 2011.

[4] Kavanaugh ML, Anderson RM. Contraception and beyond: The health benefits of services provided at family planning centers. 2013. Available at https://www.guttmacher.org/sites/default/files/pdfs/pubs/health-benefits.pdf.

[5] Michelle JH, Amanda MK, Terri-Ann T, Ushma DU. Interventions to prevent unintended and repeat pregnancy among young people in low- and middle-income countries: a systematic review of the published and gray literature. J Adolesc Health. 2016:58–515. 10.1016/j.jadohealth.2016.04.021 27562452

[6] Sedgh G, Ashford LS, Hussain R: Unmet need for contraception in developing countries: examining women’s reasons for not using a method. New York: Guttmacher Institute 2016, 2:2015–2016.

[7] Darroch JE, Audam S, Biddlecom A: Adding it up: investing in contraception and maternal and newborn health, supplementary tables. New York, NY*:* The Guttmacher Institute 2017.

[8] John S, William W: The effects of family planning and other factors on fertility, abortion, miscarriage, and stillbirths in the Spectrum model. BMC Public Health 775 *Page* 44 *of* 15 2017, 17(4). 10.1186/s12889-017-4740-7 PMC5688432 29143644

[9] Tizta TD: Why family planning matters for maternal deaths and child survival. 2017.

[10] Adedini SA, Odimegwu C, Imasiku EN, Ononokpono DN (2015). Unmet need for family planning: implication for under-five mortality in Nigeria. J Health Popul Nutr.

[11] Bradley SE, Croft TN, Fishel JD, Westoff CF: Revising unmet need for family planning. 2012.

[12] MacQuarrie K: Unmet need for family planning among young women: levels and trends. 2014.

[13] Ajmal S, Idris A, Ajmal B. Factors affecting contraceptive use and unmet need among currently married women in Afghanistan: further analysis of the Afghanistan demographic and health survey. J Glob Health Rep. 2018:2.

[14] United Nations Department of Economic and Social Affairs, population Division**. Meeting Demand for Family Planning. Population facts.** *World Contraceptive Use* 2013.

[15] FDRE: Ethiopian demographic and health survey 2016.

[16] Tegegn M, Arefaynie M, Tiruye TY (2017). Unmet need for modern contraceptives and associated factors among women in the extended postpartum period in Dessie town, Ethiopia. Contracept Reprod Med.

[17] Mota K, Reddy S, Getachew B (2015). Unmet need of long-acting and permanent family planning methods among women in the reproductive age group in shashemene town, Oromia region, Ethiopia: a cross sectional study. BMC Womens Health.

[18] Biadgie A, Nigusie A, Handebo S (2019). Prevalence and associated factors of unmet need for family planning among married women in rural communities of Gonji Kollela District, north West Ethiopia: cross-sectional study. J Fam Med Forecast.

[19] Chafo K, Doyore F (2014). Unmet need for family planning and associated factors among currently married women in Misha District, southern Ethiopia: a cross sectional study. J Women’s Health Care.

[20] Dejenu G, Ayichiluhm M, Abajobir AA: Prevalence and associated factors of unmet need for family planning among married women in Enemay District, Northwest Ethiopia: A Comparative Cross-Sectional Study. Global Journal of Medical Research 2013.

[21] Deyessa N, Argaw A. Intimate partner violence and unmet need for contraceptive use among Ethiopian women living in marital union. Ethiop J Health Dev. 2018;32(3).

[22] Gebre G, Birhan N, Gebreslasie K. Prevalence and factors associated with unmet need for family planning among the currently married reproductive age women in Shire-Enda-Slassie, Northern West of Tigray, Ethiopia 2015: a community based cross-sectional study. Pan Afr Med J. 2016;23(1). 10.11604/pamj.2016.23.195.8386 PMC4907757 27347284

[23] Genet E, Abeje G, Ejigu T (2015). Determinants of unmet need for family planning among currently married women in Dangila town administration, Awi zone, Amhara regional state; a cross sectional study. Reprod Health.

[24] Hailemariam A, Haddis F (2011). Factors affecting unmet need for family planning in southern nations, nationalities and peoples region, Ethiopia. Ethiop J Health Sci.

[25] Mekonnen W, Worku A (2011). Determinants of low family planning use and high unmet need in Butajira District**, South Central Ethiopia**. Reprod Health.

[26] Shifa GT, Kondale M. High unmet need for family planning and factors contributing to it in southern Ethiopia: A community based cross-sectional study. Glob J Med Res. 2014;14(2):20–32.

[27] Worku SA, Ahmed SM, Mulushewa TF (2019). Unmet need for family planning and its associated factor among women of reproductive age in Debre Berhan town, Amhara, Ethiopia. BMC Res notes.

[28] Yibrah HG, Gabriel TW (2018). Explaining unmet need for family planning in rural Tigrai**, Ethiopia**. J Contracept Stud.

[29] Tessema AL, Bishaw MA, Bunare TS (2015). Assessment of the magnitude and associated factors of unmet need for family planning among women of reproductive age group with disabilities in Bahir Dar City, Amhara region, north West Ethiopia. Open J Epidemiol.

[30] Kidane G, Brhane G, Abebaw G, Hailay S, Gizienesh K, Mebrahtu A (2018). Unmet need for modern contraception and associated factors among reproductive age group women in Eritrean refugee camps, Tigray, North Ethiopia: a cross-sectional study. BMC Res Notes.

[31] Moher D, Liberati A, Tetzlaff J, Altman DG, Group P (2009). Reprint—preferred reporting items for systematic reviews and meta-analyses: the PRISMA statement. Phys Ther.

[32] Munn Z, Moola S, Lisy K, Riitano D: The Joanna Briggs institute reviewers’ manual 2014. The systematic review of prevalence and incidence data Adelaide*,* SA*:* The Joanna Briggs Institute 2014.

[33] Higgins JP, Thompson SG, Deeks JJ, Altman DG (2003). Measuring inconsistency in meta-analyses. Bmj.

[34] Begg CB, Mazumdar M. Operating characteristics of a rank correlation test for publication bias. Biometrics. 1994:1088–101. 7786990

[35] Egger M, Smith GD, Schneider M, Minder C (1997). Bias in meta-analysis detected by a simple, graphical test. Bmj.

[36] Ashenafi G: Assessment of unmet need for family planning and factors influencing modern contraceptive utilization among women of reproductive age group in Girar Jarso District, North Shoa Zone, Oromia National Regional State, Ethiopia. Addis Ababa University 2011.

[37] Mihret N (2008). Determinants of unmet need for contraception among currently married couples in west belessa woreda.

[38] Molla G, Belete H. Unmet need for family planning and its determinants among currently married women in Kobbo woreda, North-East of Amhara. Ethiop J Reprod Health. 2011;5(1).

[39] Sahele S (2003). Assessment of the magnitude and determinants of unmet need for family planning among currently married women in urban and Periurban Community in Hawassa, southern Ethiopia. Ethiop J Health Sci.

[40] Antenane K: Attitudes toward family planning and reasons for nonuse among women with unmet need for family planning in Ethiopia. Calverton, Maryland USA*:* ORC Macro 2002.

[41] CSACE I: Ethiopia demographic and health survey 2016. Addis Ababa, Ethiopia, and Rockville*,* Maryland, USA*:* CSA and ICF 2016.

[42] Eregata GT, Hailu A, Memirie ST, Norheim OF. Measuring progress towards universal health coverage: national and subnational analysis in Ethiopia. BMJ Global Health. 2019;4(6). 10.1136/bmjgh-2019-001843 PMC6861121 31798996

[43] Khalil SN, Alzahrani MM, Siddiqui AF (2018). Unmet need and demand for family planning among married women of Abha, Aseer region in Saudi Arabia. Middle East Fertility Soc J.

[44] Begum S, Nair S, Donta B, Prakasam C (2014). Prevalence of unmet need for contraception in urban slum communities, Mumbai. Int J Reprod Contracept Obstet Gynecol.

[45] Abdel AAA, Amira O (2013). Factors affecting unmet need for family planning in eastern Sudan. BMC Public Health.

[46] Ragaa E-M, Noha E, Ghoneim M (2018). Unmet need for family planning among women in rural Egypt. Int J Commun Med Public Health.

[47] Atem BA, Philip NN, Martin NY, Marie JE, Felix E, Filbert EE, Bruno K, Enow RM (2016). Determinants of unmet need for family planning among women in urban Cameroon: a cross sectional survey in the Biyem-Assi Health District**, Yaoundé**. BMC Women’s Health.

[48] Fagbamigbe AF, Afolabi RF, Idemudia ES (2018). Demand and unmet needs of contraception among sexually active in-union women in Nigeria: distribution, associated characteristics, barriers, and program implications. SAGE Open.

[49] Silumbwe A, Nkole T, Munakampe MN, Milford C, Cordero JP, Kriel Y, Zulu JM, Steyn PS (2018). Community and health systems barriers and enablers to family planning and contraceptive services provision and use in Kabwe District, Zambia. BMC Health Serv Res.

[50] Laya K (2012). Prevalence and determinants of unmet need for family planning among women in India. Res Soc Pract Soc Sci.

