# Supplementary material for: Predictors of unmet need for family planning in Ethiopia 2019: a systematic review and meta analysis
Source: Arch Public Health. 2020 Oct 16;78:102. doi: 10.1186/s13690-020-00483-2 (PMC7566059; doi:10.1186/s13690-020-00483-2)
Supplement: Supplementary file 2 — Additional file 2. : Searching strategies applied for different searching data bases. [file 13690_2020_483_MOESM2_ESM.docx]

Additional file-2: Search strategy

| Databases | Search Terms | No of articles identified |
| --- | --- | --- |
| PubMed | ("unmet need for family planning"[MeSH Terms] OR ("unmet need"[All Fields] AND "family planning"[All Fields]) OR "contraception utilization "[All Fields] OR "unmet need"[AllFields] AND Associated[All Fields] AND factors[All Fields] OR "predictors"[All Fields]) AND ("Ethiopia"[All Fields]) | 96 |
| CINAHL (Plus with full text) | Thesaurus terms: family planning, unmet need, Ethiopia Search Terms: " unmet need for family planning *" OR contraception utilization" AND*unmet need " *"AND " associated" AND "Ethiopia (S1): filter: English, peer reviewed, exclude Medline (S2): with additional filter: Ethiopia S1 OR S2 | 67 |
| Cochrane Library | MeSH terms: unmet need for family planning (MeSH), contraception utilization (MeSH), unmet need (MeSH), Associated (MeSH) factors (MeSH), predictors (MeSH)  Search Terms: (family planning * OR contraception * OR utilization * AND(unmet need[MeSH] AND Ethiopia AND English [la] | 43 |
| Embase | Emtree terms: family planning, unmet need, Ethiopia Search 1 Terms: (family planning OR contraception * AND unmet need * AND (embase) NOT (medline) Search 2 Terms: (unmet need for family planning NEXT/1 family planning * OR contraception utilization * NEXT/1family planning * OR contraception utilization NEXT/1 phone* AND unmet need AND english:la AND [embase]/lim NOT [medline]/lim filter English only and excluded Medline | 83 |
| Grey Literature; Google & Google Scholar | Variety of key terms used from the above searches | 61 |
