# Supplementary material for: Predictors of unmet need for family planning in Ethiopia 2019: a systematic review and meta analysis
Source: Arch Public Health. 2020 Oct 16;78:102. doi: 10.1186/s13690-020-00483-2 (PMC7566059; doi:10.1186/s13690-020-00483-2)
Supplement: Supplementary file 3 — Additional file 3. : Joanna Briggs Institute Meta-Analysis of Statistics Assessment and Review Instrument (JBI-MAStARI) critical appraisal of studies. [file 13690_2020_483_MOESM3_ESM.docx]

| Sr.no | Criteria | Biadgie et al | Gebre et al | Dejenu et al | Shifa et al | Mota et al | Deyessa et al | Worku et al | Mekonnen et al | Yibrah et al | Tessema et al | Genet et al | Chafo et al | Tegegn et al | Hailemariam et al | Gebrecherkos et al |
| --- | --- | --- | --- | --- | --- | --- | --- | --- | --- | --- | --- | --- | --- | --- | --- | --- |
| 1 | Inclusion criteria clearly defended | yes | yes | yes | yes | yes | yes | yes | no | yes | no | no | yes | yes | no | yes |
| 2 | Description of study subject and setting | yes | yes | yes | yes | yes | no | yes | yes | yes | no | yes | yes | yes | yes | yes |
| 3 | Reliable and valid measure of exposure | no | no | no | no | no | no | no | no | no | yes | yes | yes | no | yes | no |
| 4 | Standard criteria for measure of objective | no | no | no | yes | yes | yes | yes | no | yes | no | no | no | yes | yes | no |
| 5 | Identification confounding factor | yes | yes | yes | yes | yes | yes | yes | yes | yes | yes | yes | yes | yes | yes | yes |
| 6 | Strategy to deal confounding variable | yes | yes | yes | yes | yes | yes | yes | yes | yes | yes | yes | yes | yes | yes | yes |
| 7 | Valid and reliable measure of outcomes | yes | yes | no | yes | yes | yes | yes | yes | no | yes | no | yes | no | yeyes | yes |
| 8 | Appropriate statistical analysis | yes | yes | yes | yes | yes | yes | yes | yes | yes | yes | yes | yes | yes | yes | yes |
| 9 | Response rate | yes | yes | yes | yes | yes | yes | yes | yes | yes | yes | yes | yes | yes | yes | yes |
| Total | | 7 | 7 | 6 | 8 | 8 | 7 | 8 | 6 | 7 | 6 | 6 | 8 | 7 | 8 | 7 |

JBI quality assessment checklist
