# Supplementary material for: Predictors of unmet need for family planning in Ethiopia 2019: a systematic review and meta analysis
Source: Arch Public Health. 2020 Oct 16;78:102. doi: 10.1186/s13690-020-00483-2 (PMC7566059; doi:10.1186/s13690-020-00483-2)
Supplement: Supplementary file 4 — Additional file 4. : Meta funnel presentation of the pooled prevalence of unmet need for family planning, Ethiopia. [file 13690_2020_483_MOESM4_ESM.docx]

Figure 3: Meta funnel presentation of the pooled prevalence of unmet need for family planning, E
